# Supplementary material for: Rapid Analysis of Residues of 186 Pesticides in Hawk Tea Using Modified QuEChERS Coupled with Gas Chromatography Tandem Mass Spectrometry
Source: Int J Environ Res Public Health. 2022 Oct 3;19(19):12639. doi: 10.3390/ijerph191912639 (PMC9565042; doi:10.3390/ijerph191912639)
Supplement: Supplementary file 1 [file ijerph-19-12639-s001.zip › ijerph-1905373-supplementary.pdf]

Supplementary Materials

Table S1 The major constituents of hawk tea processed by different methods

Major Constituents	Traditional Method	Green-Tea Method	Semi Fermented Method
Water extracts (%)	21.6	33.5	32
Caffeine (%)	0	0	0.1
Protein (%)	21.1	26.2	26.4
Total ash (%)	5.8	5.5	5.3
Tea polyphenols (%)	7.8	13	10
Total flavonoids (%)	2	2	2.1
Soluble sugar (%)	0.3	0.2	0.3
Fat (%)	2.4	4.1	3.2
Vitamin C (mg/g)	0.2	0.2	0.2

Table S2 the comparison of different adsorbent combination on recoveries and RSDs for 186 pesticides

No.	Pesticides	GCB		MWCNTs		EMR+ MWCNTs	
		Recoveries (%)	RSD	Recoveries (%)	RSD	Recoveries (%)	RSD
1	dichlorvos	71.3	13.6	71.5	7.6	73.9	11.4
2	dichlorobenzonitrile	66.6	9.7	70.9	2.7	65.8	10.9
3	biphenyl	60.3	11.4	60.4	1.2	56	16.9
4	etridiazole	53.7	15.4	55.8	14.7	58.8	10.4
5	propoxur	65	11.5	56.7	3.6	66.2	14.3
6	isoprocarb	68.7	2.8	66.4	11.2	73	5.8
7	tecnazene	54.1	9.4	59.2	2.2	52.9	8.4
8	diphenylamine	67.2	6.1	69.5	1.4	70.6	3.4
9	ethoprophos	83.7	5.1	91.9	5.5	94.7	13.7
10	chlorpropham	72.1	1.6	66.2	4.7	70.4	10.7
11	benfluralin	84.3	5.5	83.8	0.7	82.2	3.7

12	sulfotep	82.5	3.8	92.6	2.7	92.8	3.8
13	monocrotophos	79.1	8.3	83.1	2.3	77.3	10.9
14	phorate	63.4	5.8	62.8	5.2	67.2	4.1
15	alpha-BHC	36.1	10	43.1	4.2	44.6	8.6
16	dimethoate	81.9	1.8	85.8	2.4	88.1	2.7
17	simazine	64.1	5.2	63.5	8.2	67.8	7.3
18	atrazine	67.5	7.2	65.5	8	67.5	7.2
19	beta BHC	55.2	16.6	63.4	2.8	60.9	11.8
20	clomazone	88.1	1.9	98.3	3.1	93	2.5
21	propazine	65.7	2.7	63	2.3	62.3	2.1
22	gamma-BHC	84.4	6.6	73.1	4.9	86	7
23	profluralin	73	6.7	67.4	5.7	70.2	0.8
24	terbuthylazine	72.3	8	73.1	5.4	70.6	2.7
25	terbufos	82.8	6.3	72.8	3.5	82.8	6
26	fonofos	75.9	5.7	75.1	5.2	74.1	9.4
27	pronamide	87	6.1	92.5	7	93.4	5.3
28	diazinon	108.8	7	114.1	4.7	106.4	8
29	pyrimethanil	65.7	9.8	63.3	3.1	65.2	7.1
30	isazofos	90.5	2.6	93.3	1.5	96.4	2.6
31	etrimfos	108.1	6.6	113.8	2.3	101.8	2.4
32	delta-BHC	72.7	6.3	74.9	0.2	73.9	4.4
33	triallate	78.8	3.5	80	1.9	82.9	5.8
34	tebupirimfos	85.1	5.5	82	2.5	84.2	5.4
35	pirimicarb	77.9	7.8	84.1	2.2	80.1	4.3
36	iprobenfos	86.4	4.6	91.8	1.7	89.2	2.2
37	formothion	85.2	6.1	76.9	3.6	85.1	8.5
38	pentachloroaniline	54.5	7.2	62.4	5.7	56.5	9.6
39	phosphamidon	83.7	10.1	90.2	8.7	88.3	17.9
40	dichlofenthion	88.6	2.2	80.5	3.8	83.9	2.7

41	desmetryn	79.3	3.7	83.5	5.7	77.7	8.2
42	propanil	83.5	4.9	87.2	4.8	85.7	2.7
43	acetochlor	106.6	3.5	110.4	3	101.2	2.5
44	phenthoate	90.8	2.5	100.3	2.5	96	6.7
45	malaoxon	53.3	10.2	55.1	3.7	51.5	8.8
46	vinclozolin	76.5	3.8	86.2	2.4	81.1	1.7
47	parathion methyl	79.7	4	89.7	2.2	81.8	1.4
48	tolclofos methyl	98.7	1.8	85.8	2.1	94.4	2.5
49	alachlor	104.4	5.3	99.3	2.8	97.8	2.5
50	ametryn	87.1	5.5	92.5	4.9	83.8	15.6
51	metalaxyl	95.2	4.3	92.1	2.7	95.9	3.4
52	ronnel	75.3	7.9	69.2	1.8	71.4	4.1
53	prometryn	78.4	1.9	76.3	6.9	75.3	2.7
54	pirimiphos methyl	93.2	3.5	89.9	0.9	94.4	4.5
55	terbutryn	80.4	3.6	73.8	3.1	77.8	2.7
56	fenitrothion	97.4	2.1	89.2	2.9	92.8	0.8
57	ethofumesate	95.5	3.6	85.6	4.1	94.7	1.4
58	bromacil	75.7	4.4	78.6	14.2	74.9	5.2
59	phorate sulfoxide	85.6	5.2	77.2	4.1	82.7	10.4
60	malathion	116.5	1.4	114.8	3.5	112.2	1
61	dipropetryn	80.7	3.9	88.2	1.9	80.1	3.3
62	metolachlor	92.4	2.7	106.6	3.5	93.8	0.3
63	phoratesulfone	86.8	1.7	84.5	6	84.5	3.1
64	chlorpyrifos	76.1	4.5	100.1	1.9	95.6	1.5
65	thiobencarb	74.8	4.5	75.9	5.2	73.3	1.9
66	fenthion	96.6	1.5	90.5	14.7	90.9	3
67	parathion	96.4	1.7	98.4	5.8	92.9	2.7
68	isofenphos oxon	82.8	3.7	99.7	2.6	91	0.7
69	triadimefon	95.4	4.6	87.6	1.8	92.1	3.9

70	buprofezin	95.4	4.7	100.5	7.3	94.4	9.6
71	isocarbophos	109.7	4.9	94.6	3.6	104.6	6.3
72	dicofol	89.3	4.9	102	2.9	92.9	4.6
73	trichloronat	86	4.4	93.6	3.8	87	3.7
74	pirimiphos ethyl	84.4	4.6	87	2.4	82.8	3.4
75	bromophos	79.5	1.8	85	5.3	83.7	3.1
76	isofenphos methyl	92.1	4.1	94.9	3	93.5	1.7
77	fosthiazate	66	7.4	70.3	2.4	70.7	10.7
78	pendimethalin	88.9	2.5	95	6.6	87.6	3.9
79	chlorfenvinphos	110.5	1.4	99.4	3.4	106.7	1.3
80	cyprodinil	54.7	8	67.7	6.5	58.4	1.2
81	terbufos sulfone	82.5	3.2	88.5	2.3	80.4	3.3
82	fipronil	103.2	6.2	89.9	3.7	98.5	3.6
83	penconazole	72.7	6.5	80.5	1.1	74.9	5.7
84	phosfolan	55.1	7.8	62.8	16.8	59	2.3
85	isofenphos	86.5	2.3	93.1	3.6	90.1	1
86	beflubutamid	104.7	2.5	104.4	3.2	100.8	1.5
87	quinalphos	76.6	0.8	88.4	2.8	85.5	2.1
88	mephosfolan	64.8	5.7	71.2	4.2	65.4	3.2
89	procymidone	108.3	2.4	93.4	3.9	103.6	1.3
90	triadimenol	91.8	3.8	84	2.2	89.9	2.7
91	bromophos ethyl	95.6	4.7	92.1	1.9	93.5	5.4
92	methidathion	77.7	0.8	88.8	3.2	82.5	2.4
93	chlordane trans	85.4	1.7	86.2	7	87.8	5.7
94	op'-DDE	74.4	4.2	79	4.1	78.7	3.7
95	paclobutrazol	82.5	3.7	90	6.6	88.2	2.4
96	butachlor	84.4	4.7	95.9	3.5	95.3	3.8
97	fenothiocarb	78.4	2.9	84.7	3.8	81	1.5
98	ditalimfos	54.7	4.2	59.1	3.8	57.8	1.8

99	butamifos	77.6	1.2	96.1	4.6	82	4.1
100	napropamide	95	4.5	95.7	8.1	95.7	3.3
101	bromfenvinfos	86.2	4.3	92.3	4.7	89	1
102	fluorodifen	96.2	3.6	94.2	10.2	95.9	3.5
103	flutolanil	111.5	4.5	108.1	6.3	101.8	0.6
104	chlorfenson	109.2	1.7	107.8	3.7	99	3.8
105	hexaconazole	81.5	1	85.8	7.1	84.2	6.6
106	prothiofos	72.7	5.6	84.2	2.9	73.6	3.7
107	fludioxonil	94.1	6.6	96.2	4.3	92.6	6.4
108	pretilachlor	94.8	4.2	95.7	2.1	92.1	3.3
109	isoprothiolane	90.1	1.3	100.4	4.4	93.2	2.7
110	profenofos	91.6	1.8	81.4	5.9	88.7	0.4
111	pp'-DDE	74.2	3.3	74.2	2.4	74.1	2.9
112	oxadiazon	80.9	9.7	81.3	7.2	81.3	3.6
113	DEF	74.6	4.6	83.8	3.7	78.1	0.3
114	dieldrin	84.3	6.7	97	1.9	86.9	7.1
115	myclobutanil	85.2	6.2	93.4	1.9	83.3	3.7
116	op'-DDD	88.9	0.9	83.6	6.2	83.8	0.8
117	oxyfluorfen	78.6	6.2	90.4	7.7	81.8	4.5
118	bupirimate	75.6	6.4	120.7	1.4	75.5	2.3
119	kresoxim methyl	96.3	3.9	95.9	5.4	92.5	1.5
120	cyflufenamid	82.4	6	89.3	3.5	83.7	8.6
121	isoxathion	80.3	1.2	76.8	8.4	76.7	3.9
122	cyproconazole 1	91	2	92.3	3.5	92	2.4
123	fluazifop butyl	100.7	1.6	95.1	6.6	93	1.9
124	nitrofen	81.2	2.8	86.6	7.6	85.9	2.8
125	endrin	83.4	6.4	79.1	4	79.5	4.2
126	chlorobenzilate	84.5	8.6	88.7	4.2	89.1	2.3
127	fensulfothion	89.1	1.1	91.5	3.9	85.8	8.1

128	diniconazole	65.7	5.8	72.6	4.8	71	2.4
129	oxadixyl	105.6	4.1	99.5	3.9	96.6	2.6
130	pp'-DDD	87.8	1.7	84	2.6	83.8	2
131	ethion	112	2	105.7	2.8	105.4	1.9
132	op'-DDT	76.8	0.8	78.2	10.6	76.9	3.6
133	chlorthiophos	85.4	3.8	81.9	1.6	81.9	2.6
134	aclonifen	64.8	2.4	70.9	5.9	71.2	3.2
135	triazophos	86.8	1.5	90.4	6	85.7	0.2
136	famphur	89.4	1.9	89.9	7.5	88.8	2.4
137	benalaxyl	101.4	1.8	90.8	3.1	95.4	1.2
138	carbophenothion	92.9	7.2	86.7	2.9	89.4	1.1
139	trifloxystrobi	79.8	1.2	87.9	4.3	83.3	3.2
140	edifenphos	75.5	5.3	78.7	2.1	77.1	2.1
141	quinoxyfen	46.5	8	55.8	6	49.7	5.8
142	propiconazole	78	3	78.3	4.8	77.7	6.1
143	pp'-DDT	80.4	3.8	76.3	3.6	79	1.1
144	hexazinone	80.7	1.5	81.9	4.9	78.9	1.7
145	tebuconazole	75.1	5.5	76.9	3.9	75.2	2.7
146	diclofop methyl	90.3	9	101.6	4.4	92.9	1
147	piperonylbutoxide	122.8	4.4	115.7	6.9	119.7	3.4
148	epoxiconazol	73.2	5.5	79.7	3.9	77.3	2.2
149	pyridaphenthion	90.6	1.9	94.3	0.8	88	2.5
150	iprodione	87.1	4.1	86.6	5.1	82.1	1.6
151	phosmet	75.9	6.3	82.9	3.1	79	3.9
152	bifenthrin	83.3	5.1	88.8	1.6	86.6	1.6
153	EPN	80.3	1.9	78.9	1.5	78.5	3.2
154	bromopropylate	100.4	4.6	89.4	10.5	93.5	2.1
155	piperophos	85.1	5.1	90.4	3.6	90.7	2.6
156	tetramethrin	97.2	4.2	92.1	3.4	92.9	4.3

157	methoxychlor	87.9	1.6	90	2.4	87.9	3.2
158	etoxazole	88.8	1.6	88.6	5.6	86.6	3.3
159	fenamidone	92.4	4.8	86	6.8	90.5	3
160	tebufenpyrad	78.7	2.2	83.3	4.1	77.3	2.9
161	anilofos	92.4	5.3	94.6	5.8	91.8	4.2
162	bifenox	92.5	3.8	99.9	6.1	92.2	7.5
163	tetradifon	75	5	84.5	2.7	73.9	3.6
164	phosalone	86	5.5	82.6	1.9	83.3	1.6
165	leptopho	64.3	4.4	70.8	4.2	70.8	4
166	pyriproxyfen	82.6	4.3	81.3	2.9	80.6	1.4
167	iambda cyhalothrin	95.5	4.6	96.1	2.6	92.5	4
168	mefenacet	89.5	1.1	83.5	3.5	85.4	1.5
169	acrinathrin	76.2	5.9	85.5	4.1	75.9	2.7
170	pyrazophos	75.9	6.1	80.2	5.1	77.7	7
171	fenarimol	77.8	1.6	81.3	5.8	79.3	3.7
172	azinphos ethyl	86.9	2	82.9	4.8	80.8	7.1
173	permethrin 1	91.8	3.8	86.4	6.6	88.4	3.6
174	coumaphos	105	2.5	104.9	6.1	96.4	4.6
175	fluquinconazole	94.9	6	89.9	2.5	87	6.5
176	pyridaben	74.3	4.7	76.2	6.5	73.7	3.2
177	dioxathion	66.9	7.9	66.6	2.2	74.6	4.4
178	fenbuconazole	82.8	2	79.4	1.4	85.5	5
179	cyfluthrin	83.5	5.5	85.3	8.7	82.6	1.5
180	cypermethri	75.6	5.7	86.3	4.7	78.9	3.5
181	boscalid	67.4	5.5	71	0.7	72.8	3.5
182	flucythrinate	96.2	4.8	93.9	2.7	92.2	3.1
183	fenvalerate	116.5	4.7	114.1	2.3	119.1	3.9
184	fluvalinate	71.4	2	77.6	4.2	76	3.7
185	difenoconazole	76.4	5.3	82.1	5.2	77.7	3.6

186	deltamethrin	75.4	4.1	73.1	2.4	73.1	5.8

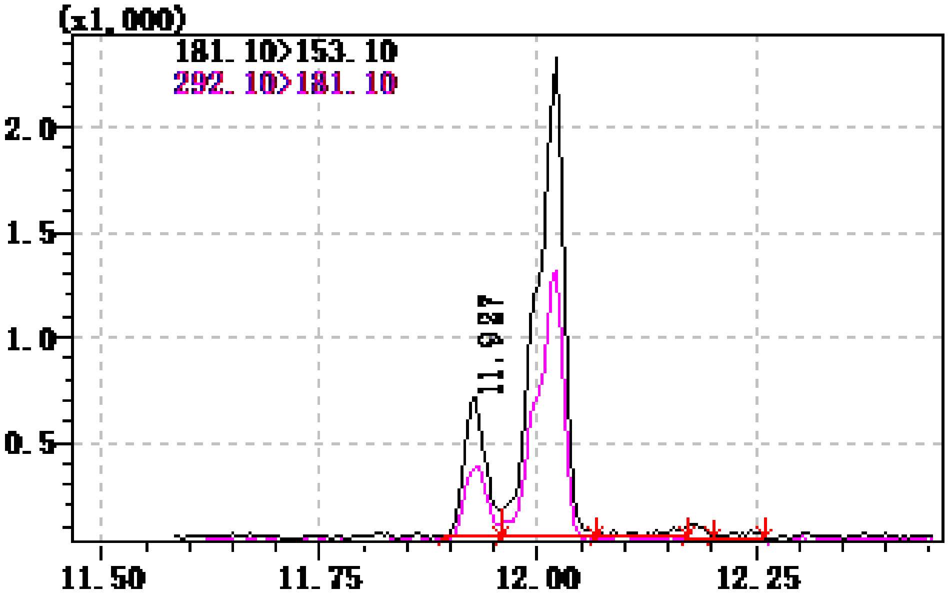

Figure S1 Chromatogram of etrimfos in the mixed solvent of acetonitrile and toluene

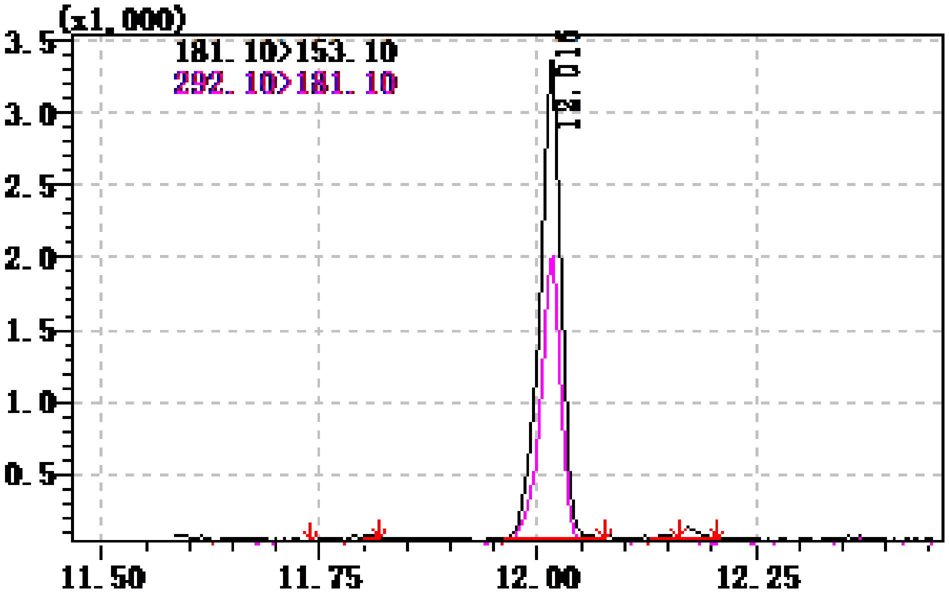

Figure S2 Chromatogram of etrimfos in ethyl acetate

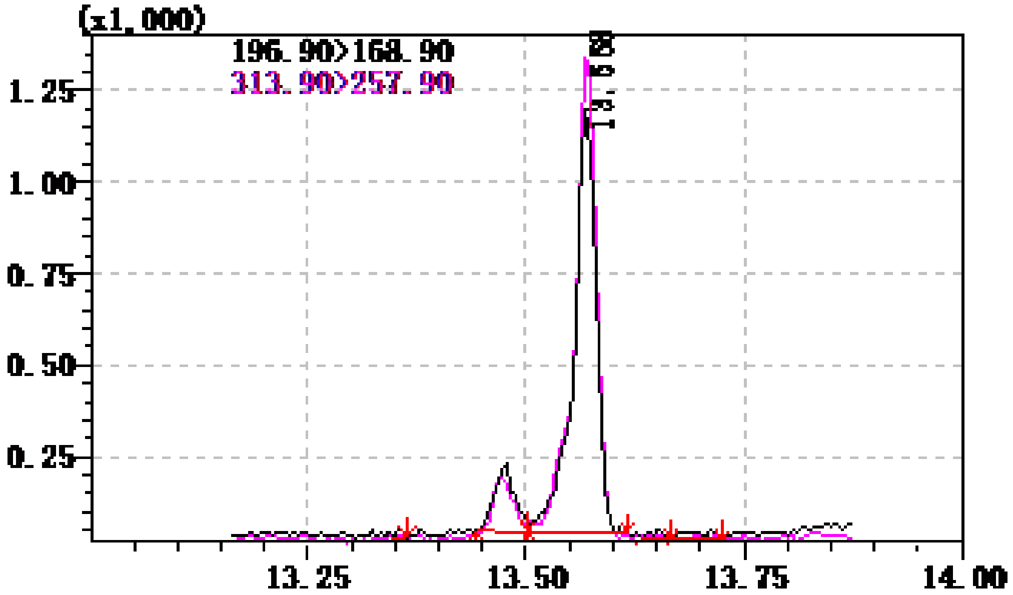

Figure S3 Chromatogram of chlorpyrifos in the mixed solvent of acetonitrile and toluene

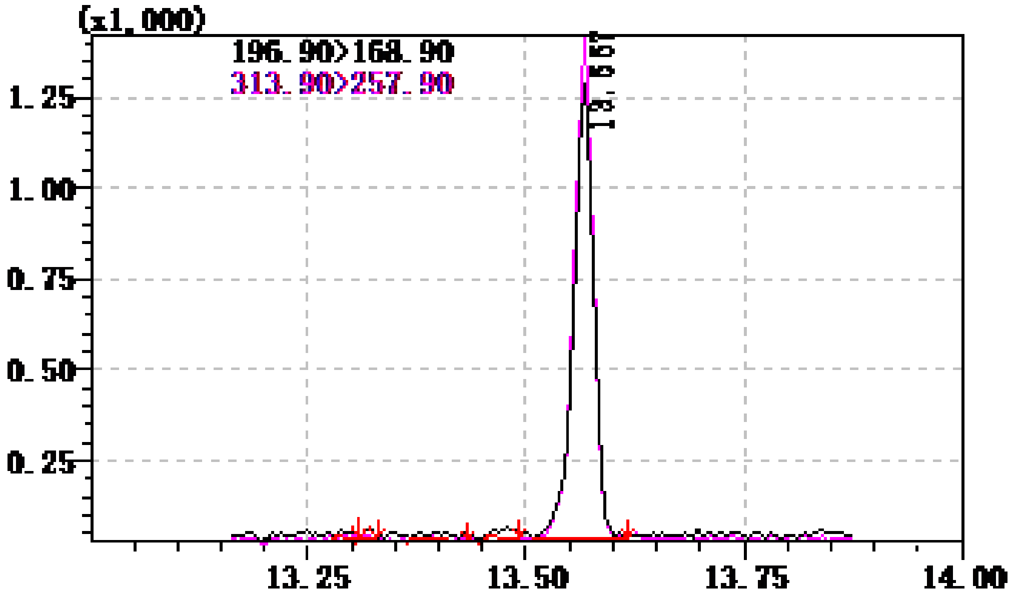

Figure S4 Chromatogram of chlorpyrifos in ethyl acetate

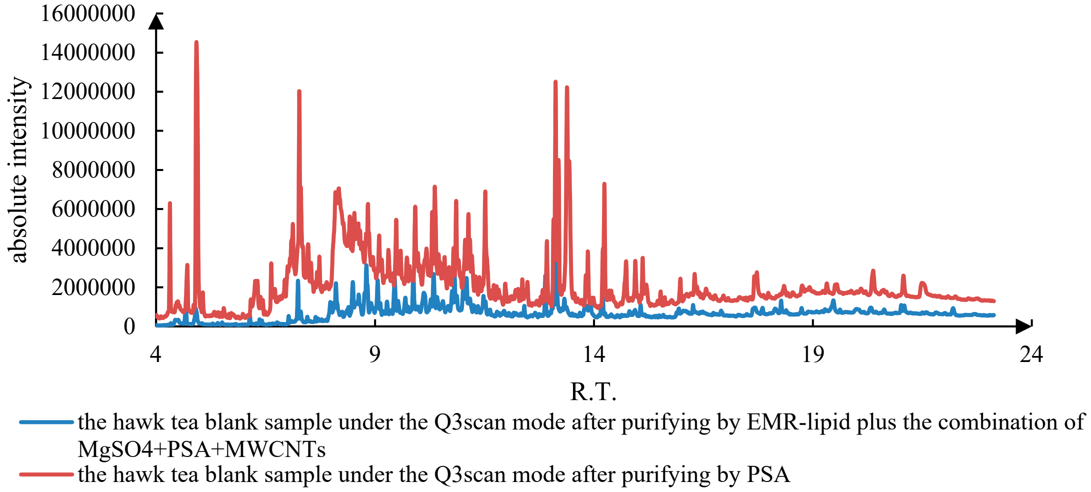

Figure S5 The hawk tea blank sample under the Q3scan mode after purification by different materials.
